# Efficacy and safety of dupilumab in moderate-to-severe atopic dermatitis with stage 3–5 chronic kidney disease: a retrospective real-world study

**DOI:** 10.3389/fmed.2026.1906235

**Published:** 2026-09-14

**Authors:** Jiali Wei, Yiqi Huang

**Affiliations:** 1 Department of Dermatology, Shaoxing Second Hospital, Shaoxing, Zhejiang, China; 2 Department of Nephrology, Shaoxing Second Hospital, Shaoxing, Zhejiang, China

**Keywords:** atopic dermatitis, chronic kidney disease, dupilumab, efficacy, safety

## Abstract

**Aims:**

To evaluate dupilumab efficacy and safety in stage 3–5 chronic kidney disease (CKD) patients with moderate-to-severe atopic dermatitis (AD).

**Methods:**

This retrospective study included 29 such patients receiving dupilumab who completed 12 and 24 weeks of follow-up. Assessments included Eczema Area and Severity Index (EASI), Pruritus Numeric Rating Scale (*N*RS), Sleep NRS, Dermatology Life Quality Index (DLQI), and Clinical Frailty Scale (CFS), eosinophils, interleukin-10 (IL-10), regulatory T cell (Treg) proportions, and renal function [estimated glomerular filtration rate (eGFR), serum creatinine, and urine protein-to-creatinine ratio (UPCR)]. Adverse events were recorded.

**Results:**

At week 12, mean EASI score decreased from 34.94 ± 7.96 at baseline to 19.31 ± 6.09 (44.7% reduction), with 76.0% of patients achieving EASI-50. Pruritus NRS decreased from 7.72 ± 1.75 to 3.72 ± 1.75 (51.8% reduction), and 88.0% achieved a ≥ 4-point improvement. DLQI improved by 52.1%, and 100% of patients achieved a ≥ 4-point improvement. CFS score decreased by 17.5%. Eosinophil counts decreased by 25.0%, while IL-10 and Treg proportions increased by 49.9% and 50.8%, respectively. At week 24, EASI-50, EASI-75, and EASI-90 response rates were 100%, 76.9%, and 7.7%, respectively; 100% of patients achieved ≥4-point improvements in both pruritus NRS and DLQI. IL-10 and Treg continued to rise, and CFS score was significantly reduced from baseline (*P* = 0.001). Renal function parameters remained stable throughout the 24-week follow-up: mean eGFR showed a minimal decline from 37.93 ± 14.88 to 36.39 ± 14.77 mL/min/1.73 m^2^ at week 24 (*P* = 0.073), serum creatinine and UPCR also showed no significant changes (*P* = 0.130 and *P* = 0.069, respectively). Only two mild-to-moderate adverse events (injection-site reaction and conjunctivitis) occurred, both resolved with symptomatic management. No treatment discontinuation, death, or worsening of renal function was reported.

**Conclusion:**

Dupilumab effectively improves skin lesions, pruritus, sleep, quality of life, and frailty, while showing changes consistent with regulatory immune modulation in patients with stage 3–5 CKD and moderate-to-severe AD, with a favorable safety profile. Renal impairment alone should not be considered an absolute contraindication, although patients with advanced CKD require careful monitoring.

## 1 Introduction

Atopic dermatitis (AD) is a chronic inflammatory skin disease driven by intense pruritus and eczematous lesions. Its prevalence has been rising across all age groups (1, 2). Globally, AD affects 2%–10% of the adult population (3). Beyond its cutaneous manifestations, AD is increasingly recognized to be associated with systemic comorbidities, among which CKD stands out as particularly consequential. Epidemiological evidence, including a nationwide population based cohort analysis, indicates that individuals with AD face a substantially elevated risk of developing CKD (4). Conversely, patients with CKD exhibit a higher than expected prevalence of AD, as suggested by a bidirectional Mendelian randomization study (5).

Managing moderate to severe AD in the setting of advanced CKD presents formidable challenges. Refractory pruritus, whether arising from uremic toxin accumulation or from active AD, afflicts a large proportion of CKD patients (6). It severely impairs quality of life and sleep and correlates with increased mortality (7). Traditional systemic immunosuppressants such as cyclosporine and methotrexate are often contraindicated or poorly tolerated in CKD due to nephrotoxicity, myelosuppression, and heightened infection risk (8, 9). There is thus an urgent unmet need for therapies that are both efficacious against AD and intrinsically safe in the context of renal impairment.

Dupilumab is a fully human IgG4 monoclonal antibody that targets the interleukin-4 receptor *α* subunit (IL-4R*α*) and concurrently blocks interleukin-4 (IL-4) and interleukin-13 (IL-13) signaling, thereby suppressing type 2 inflammation (10, 11). Multiple phase 3 trials and real-world studies have established its long-term efficacy and safety in children, adolescents, and adults with AD (12–14). Unlike conventional immunosuppressants, dupilumab is not renally metabolized, lacks nephrotoxicity, and exhibits few drug–drug interactions, attributes that confer a unique therapeutic advantage in patients with CKD (15, 16). A small number of recent studies have reported favorable efficacy and safety of dupilumab in CKD patients with AD (17); however, large-scale real-world evidence focusing specifically on those with stage 3–5 CKD and moderate-to-severe AD remains scarce.

To address this gap, the present study retrospectively evaluates the efficacy and safety of dupilumab in this understudied population, aiming to provide clinical reference for managing a challenging patient group at the intersection of advanced CKD and severe AD.

## 2 Materials and methods

### 2.1 Study design and participants

This single-center retrospective study included patients with stage 3–5 CKD and moderate-to-severe AD who received dupilumab treatment at the Department of Dermatology, Shaoxing Second Hospital, between January 2022 and June 2025. Eligible patients met the following criteria: an estimated glomerular filtration rate (eGFR) < 60 mL/min/1.73 m^2^ (18); a diagnosis of AD according to the Hanifin and Rajka criteria (19); age ≥ 18 years; a baseline Eczema Area and Severity Index (EASI) score ≥ 16; and prior failure of at least one systemic therapy. Patients were excluded if they had a malignancy, active tuberculosis, severe hepatic or renal dysfunction, immunodeficiency, or a known allergy to any component of dupilumab. Of the 42 patients who initiated dupilumab, 13 were excluded from the final analysis due to follow-up <24 weeks (*n* = 9) or loss to follow-up (*n* = 4). Their characteristics are summarized in Supplementary Table S1. After applying these criteria, 29 patients were eventually enrolled (Supplementary Figure S1). The study was conducted in accordance with the ethical principles of the Declaration of Helsinki. Approval was obtained from the Ethics Committee of Shaoxing Second Hospital, and written informed consent was secured from all participants.

### 2.2 Treatment regimen

All patients received subcutaneous injections of dupilumab (Sanofi, France). The initial dose was 600 mg, followed by 300 mg every 2 weeks. Follow-up visits were scheduled every 4 weeks. The decision to continue treatment was based on clinical efficacy and tolerability.

### 2.3 Data collection and efficacy assessment

Demographic and clinical data were retrospectively collected from the electronic medical record system, including age, sex, age at onset of AD, clinical phenotype, comorbidities (hypertension, cardiovascular disease, diabetes mellitus, chronic obstructive pulmonary disease, neurological disorders, etc.), and prior systemic therapies for AD (e.g., glucocorticoids, immunosuppressants, phototherapy, traditional Chinese medicine).

The following scoring systems were applied at baseline, week 12, and week 24 (or at the last follow-up visit): EASI, Pruritus Numeric Rating Scale (*N*RS), Sleep Disturbance NRS, Dermatology Life Quality Index (DLQI), and Clinical Frailty Scale (CFS). These instruments assessed five independent domains: objective skin lesions, pruritus intensity, sleep loss, quality of life, and overall frailty status. Frailty was assessed using the CFS, a well-validated bedside instrument that captures global functional status, which is particularly relevant in patients with advanced chronic kidney disease who are prone to sarcopenia and inflammatory wasting.

To evaluate renal function, immune responses, and inflammatory changes, eGFR (calculated using the CKD-EPI equation), serum creatinine, urine protein-to-creatinine ratio (UPCR), peripheral blood eosinophil count, interleukin-10 (IL-10) level, and regulatory T-cell (Treg) proportion were measured at baseline, week 12, and week 24 (or at the last follow-up visit). Concomitant use of topical corticosteroids, calcineurin inhibitors, emollients, and oral antihistamines was permitted as needed, and their use was recorded at each visit.

Adverse events occurring during treatment were recorded, including injection-site reactions, ocular reactions (e.g., conjunctivitis), gastrointestinal symptoms, cutaneous reactions (e.g., facial erythema, folliculitis), as well as influenza and other respiratory tract infections.

### 2.4 Statistical analysis

Statistical analyses were performed using SPSS version 27.0, and graphs were generated with GraphPad Prism 7.0. Categorical variables were expressed as counts (percentages). Continuous data were first tested for normality using the Shapiro–Wilk test. Normally distributed data were presented as mean ± standard deviation (SD), while non-normally distributed data were presented as median (interquartile range, IQR).

Paired comparisons were conducted between baseline and week 12, as well as between baseline and week 24. For each paired dataset, if the differences followed a normal distribution, a paired-sample t-test was applied; otherwise, the Wilcoxon signed-rank test was used. All tests were two-sided, and a significance level of *α* = 0.05 was set; *P* < 0.05 was considered statistically significant.

## 3 Results

### 3.1 Clinical demographic characteristics and features

A total of 29 patients with stage 3–5 CKD and moderate-to-severe AD were enrolled and completed 24 weeks of follow-up (baseline characteristics in Table 1). The mean age was 55.7 years, and 68.97% were male. Most patients had stage 3 CKD (68.97%), with chronic glomerulonephritis (41.38%) being the leading etiology, followed by diabetic nephropathy (27.59%), hypertensive nephrosclerosis (17.24%), and polycystic kidney disease (13.79%). Median CKD duration was 4.53 (2.05, 8.11) years; the three stage 5 patients were managed conservatively without dialysis. Hypertension (68.97%), cardiovascular disease (58.62%), and diabetes (41.34%) were the predominant comorbidities, with concomitant use of renin-angiotensin system inhibitors (62.09%), SGLT2 inhibitors (37.93%), and diuretics (17.24%). Clinically, the generalized phenotype was most common (34.48%). At baseline, patients exhibited severe skin lesions (mean EASI 34.90), marked pruritus (median NRS 8.0), substantially impaired quality of life (mean DLQI ∼16), and mild frailty (median CFS 4.0). Laboratory findings showed mildly elevated eosinophil counts (0.64  ×  10^9^/L) and low-normal levels of IL-10 (14.98 pg/mL) and Treg proportion (4.35%).

**Table 1:** Clinical characteristics.

Clinical characteristics	Value (*n* = 29)
Sex, male, *n* (%)	20 (68.97%)
Age, years, Mean ± SD	55.73 ± 4.28
Age at onset of AD, years, Mean ± SD	42.31 ± 4.18
Chronic Kidney Disease staging, *n* (%)	
Stage 3 (eGFR 30 −59 mL/min/1.73m^2^)	20 (68.97)
Stage 4 (eGFR 15–29 mL/min/1.73m^2^)	6 (20.69)
Stage 5 (eGFR <15 mL/min/1.73m^2^)	3 (10.34)
CKD duration, years, *M* (Q_1_, Q_3_)	4.53 (2.05, 8.11)
CKD etiology	
Chronic glomerulonephritis	12 (41.38%)
Diabetic nephropathy	8 (27.59%)
Hypertensive nephrosclerosis	5 (17.24%)
Polycystic kidney disease	4 (13.79%)
Clinical phenotype, *n* (%)	
Generalized type	10 (34.48%)
Head-and-neck type	7 (24.14%)
Hand eczema type	5 (17.24%)
Prurigo type	4 (13.79%)
Erythrodermic type	3 (10.35%)
Nephrology medications	
Renin-angiotensin system inhibitors	18 (62.07%)
Sodium-glucose cotransporter-2 inhibitors	11 (37.93%)
Diuretics	5 (17.24%)
Comorbidities, *n* (%)	
Hypertension	20 (68.97%)
Cardiovascular disease	17 (58.62%)
Diabetes mellitus	12 (41.34%)
Chronic obstructive pulmonary disease	7 (24.14%)
Neurological disorders	8 (27.59%)
Previous systemic therapies, *n* (%)	
Antihistamines	21 (72.41%)
Glucocorticoids	15 (51.72%)
Traditional Chinese medicine	10 (34.48%)
Immunosuppressants	9 (31.03%)
Phototherapy	6 (20.69%)
EASI, Mean ± SD	34.90 ± 7.88
Pruritus NRS, M (Q_1_, Q_3_)	8.00 (7.00,9.00)
Sleep NRS, M (Q_1_, Q_3_)	7.00 (6.00,8.00)
DLQI, Mean ± SD	16.38 ± 4.98
CFS, M (Q_1_, Q_3_)	4.00 (4.00,5.00)
Eosinophil, ×10^9^/L, Mean ± SD	0.64 ± 0.20
IL-10, pg/mL, Mean ± SD	14.98 ± 1.88
Treg proportion, %, Mean ± SD	4.35 ± 0.59

EASI, Eczema Area and Severity Index; NRS, Numeric Rating Scale; DLQI, Dermatology Life Quality Index; CFS, Clinical Frailty Scale; Treg, Regulatory T cell; IL-10, Interleukin-10.

### 3.2 Efficacy assessment

At week 12, all clinical endpoints showed significant improvement (Table 2, Supplementary Figure S2). The EASI score decreased by approximately 45% from baseline, with 76.0% of patients achieving EASI-50. Pruritus NRS fell by more than 50%, and 88.0% of patients attained a ≥ 4-point improvement. Sleep NRS and DLQI also markedly improved; all patients achieved a ≥ 4-point DLQI reduction. The CFS score decreased relative to baseline, suggesting an amelioration of frailty status. Laboratory findings revealed a 25.0% decline in eosinophil counts, together with increases of about 50% in both IL-10 and Treg proportion.

**Table 2:** Changes in clinical scores and laboratory parameters in AD patients treated with dupilumab.

Time point	EASI		Pruritus NRS		Sleep NRS		DLQI		CFS		EOS, ×10^9/L		IL-10, pg/mL		Treg, %	
	Mean ± SD	*P*	M (Q₁, Q₃)	*P*	M (Q₁, Q₃)	*P*	Mean ± SD	*P*	M (Q₁, Q₃)	*P*	Mean ± SD	*P*	Mean ± SD	*P*	Mean ± SD	*P*
Baseline	34.90 ± 7.88		8.00 (7.00, 9.00)		7.00 (6.00, 8.00)		16.38 ± 4.98		4.00 (4.00, 5.00)		0.64 ± 0.20		14.98 ± 1.88		4.35 ± 0.59	
Week 12	19.31 ± 6.09	0.043*	3.00 (3.00, 5.00)	0.000*	3.00 (2.00, 4.00)	0.000*	7.86 ± 3.88	0.000*	4.00 (3.00, 4.00)	0.000*	0.48 ± 0.10	0.000*	22.45 ± 2.42	0.000*	6.56 ± 0.63	0.000*
Week 24	9.38 ± 4.87	0.000**	1.00 (0.00, 2.00)	0.000**	1.00 (0.00, 2.00)	0.000**	2.83 ± 2.17	0.000**	2.00 (1.00, 3.00)	0.001**	0.40 ± 0.08	0.000**	28.27 ± 2.48	0.000**	7.93 ± 0.71	0.000**

EASI, Eczema Area and Severity Index; NRS, Numeric Rating Scale; DLQI, Dermatology Life Quality Index; CFS, Clinical Frailty Scale; Treg, Regulatory T cell; IL-10, Interleukin-10; EOS, Eosinophil.

* *p* compared with baseline.

** *p* compared with baseline.

By week 24, the therapeutic benefit had deepened further (Table 2, Supplementary Figure S2). The EASI score continued to decline, with an EASI-75 response rate of 76.9% and the emergence of EASI-90 responses (7.7%). All patients achieved a ≥ 4-point improvement in both pruritus and DLQI, and sleep NRS scores fell to very low levels. The CFS score was significantly reduced from baseline (*P* = 0.001). Immunological parameters continued to improve: eosinophils further decreased, whereas IL-10 and Treg proportions kept rising, indicating progressive restoration of regulatory immune function. The absolute changes (Δ) and their 95% confidence intervals (CIs) for all endpoints are detailed in Supplementary Table S2, offering a quantitative measure of effect sizes. At week 12, the mean reductions from baseline were −15.60 (95% CI, −19.17 to −12.02) for EASI, −4.00 (95% CI, −5.01 to −2.99) for pruritus NRS, and −8.54 (95% CI, −10.73 to −6.34) for DLQI; corresponding values at week 24 were −25.52 (95% CI, −28.83 to −22.21), −6.72 (95% CI, −7.40 to −6.05), and −13.55 (95% CI, −15.25 to −11.85), respectively. The CFS also showed significant reductions (Δ −0.79, 95% CI −1.29 to −0.29 at week 12; Δ −2.34, 95% CI −2.89 to −1.80 at week 24). All these CIs excluded zero, confirming the robustness of the observed clinical benefits. In parallel, renal function parameters exhibited minimal fluctuations, with ΔeGFR of −1.55 (95% CI, −2.63 to −0.47) and ΔUPCR of −0.02 (95% CI, −0.05 to 0.01) at week 24, consistent with the stability described above.

Renal function remained stable throughout the 24-week follow-up (Table 3). Mean eGFR showed a minimal decline from 37.93 ± 14.88 mL/min/1.73 m^2^ at baseline to 36.39 ± 14.77 at week 24 (*P* = 0.073); serum creatinine increased slightly from 154.90 (124.50, 223.80) to 160.40 (131.20, 255.30) µmol/L (*P* = 0.130); and UPCR changed from 0.802 ± 0.345 to 0.765 ± 0.340 g/g (*P* = 0.069). None of these changes reached statistical significance (all *P* > 0.05). No patient required renal replacement therapy or developed acute kidney injury; the three stage 5 patients were managed conservatively without dialysis.

**Table 3:** Changes in renal function in AD patients treated with dupilumab.

Time point	eGFR (mL/min/1.73m^2^)		Serum creatinine (µmol/L)		UPCR (g/g)	
	Mean ± SD	*P*	Mean ± SD	*P*	*M* (Q_1_, Q_3_)	*P*
Baseline	37.93 ± 14.88		154.90 (124.50, 223.80)		0.802 ± 0.345	
Week 12	37.90 ± 14.97	0.920*	154.00 (125.50, 224.10)	0.991*	0.774 ± 0.328	0.063*
Week 24	36.39 ± 14.77	0.073**	160.40 (131.20, 255.30)	0.130**	0.765 ± 0.340	0.069**

eGFR, estimated Glomerular Filtration Rate; UPCR, urine protein-to-creatinine ratio.

* *p* compared with baseline.

** *p* compared with baseline.

### 3.3 Safety assessment

A total of 29 patients were included in the safety analysis during the treatment and follow-up period. Two patients experienced adverse events, including injection-site reactions (redness, pain) and ocular symptoms (conjunctivitis). All events were mild to moderate and resolved with symptomatic management. No patient discontinued treatment due to adverse events. No serious adverse events such as severe allergic reactions, hepatic or renal impairment, malignancy, or active tuberculosis infection were observed. No deaths occurred in this study.

## 4 Discussion

This retrospective study of 29 patients with stage 3–5 CKD and moderate-to-severe AD provides real-world evidence that dupilumab is effective, well tolerated, and safe in this understudied population. At both 12 and 24 weeks, we observed significant improvements across five clinically relevant domains: skin lesions, pruritus, sleep quality, quality of life, and frailty. Notably, eosinophil counts decreased, whereas levels of IL-10 and proportions of Treg progressively increased. Taken together, these findings suggest that renal impairment alone should not be viewed as an absolute barrier to dupilumab therapy in appropriately selected patients.

When compared with similar studies, our findings broadly align with recent real-world evidence, despite differences in sample size, CKD stage distribution, and efficacy measures. Peng et al. reported a 68.5% reduction in EASI and a 66.7% EASI-75 response rate at week 16 in 18 patients with CKD and AD (17). In our study, the EASI-75 rate at week 24 reached 76.9%, a modestly higher figure. This difference likely reflects the distinct CKD severity profiles between the two cohorts: only 10.3% of our patients had stage 5 CKD, whereas Peng et al. included a larger proportion of stages 4–5 disease (17). More importantly, Wang et al. provided a complementary perspective (20). Their study enrolled not only 12 CKD patients with AD but also 10 patients with isolated uremic pruritus. The two groups showed no significant difference in the proportion achieving a ≥ 4-point improvement in PP-NRS or a 5D-IS score ≤10, suggesting that dupilumab's antipruritic effect may be independent of an AD diagnosis and more broadly applicable to type 2 inflammation-driven itch in CKD. In our cohort, the proportion of patients achieving a ≥ 4-point reduction in pruritus NRS rose from 88.0% at week 12 to 100% at week 24, closely matching the 86.9–93.7% range reported in the literature. This consistency further supports the notion that IL-4 and IL-13 signalling play a shared, and possibly central, role across distinct forms of CKD-associated pruritus.

From a mechanistic perspective, dupilumab targets the IL-4 receptor *α* subunit, thereby blocking IL-4 and IL-13 signalling and suppressing type 2 inflammation (21). The marked reduction in eosinophil counts observed in our study confirms this well-established downstream effect. More revealing, however, was the concurrent rise in IL-10 levels and Treg proportions. Patients with AD are known to exhibit deficits in the number or function of peripheral blood Treg cells, and these deficits correlate with disease severity (22). IL-4 can directly inhibit Treg proliferation and reduce their expression of CTLA-4, thereby weakening their suppression of Th2-type inflammation. Against this background, the progressive increase in Treg proportions from baseline in our study aligns with several recent immunophenotypic reports. For instance, a prospective study of 25 AD patients found that after six months of dupilumab treatment, circulating Th2 cells decreased by approximately 0.55-fold, whereas Treg cells increased by an average of 27%, accompanied by a roughly 1.96-fold rise in IL-10 secretion (23). Another study demonstrated that dupilumab treatment rapidly modulates skin-homing CD4+ T-cell functions, with significant effects on CLA + and CCR4 + populations observed as early as 4 weeks after initiation (24), suggesting dynamic regulation of tissue-homing T cells during treatment. A cross-sectional study by Čelakovská et al. also confirmed that plasma IL-10 levels were significantly higher in dupilumab-treated patients than in controls (25).

These changes in Treg and IL-10 carry particular pathophysiological relevance in the context of CKD. Chronic kidney disease itself is often accompanied by Treg dysfunction and a state of chronic low-grade inflammation (26). Renal failure can impair the immunosuppressive function of Treg, and dialysis treatment may further affect Treg number and phenotype (27, 28). In our study, both Treg proportion and IL-10 level continued to rise from week 12 to week 24 (*p* < 0.001 for both), indicating that longer treatment yields progressively deeper immunomodulatory benefits. This effect may be especially advantageous for the persistent inflammatory state that characterises CKD. IL-10 not only restrains Th1, Th2, and Th17 responses but also promotes immune tolerance (29), potentially alleviating the systemic microinflammatory state seen in some CKD patients (30). Our findings extend the immunomodulatory profile of dupilumab from the skin to the peripheral blood and, importantly, provide the first direct immunological evidence in the specific population of patients with both CKD and AD. However, it is important to acknowledge that the observational design of this study limits causal inference, and the observed changes in Treg and IL-10 may reflect improvement in systemic inflammation accompanying disease control rather than a direct immunological mechanism of dupilumab.

Another unique finding of this study is the sustained improvement in frailty status with dupilumab treatment. Frailty is highly prevalent in patients with CKD and serves as an important predictor of hospitalisation, dialysis-related complications, and mortality (31). Its pathophysiology involves multiple factors, including chronic low-grade inflammation, immune senescence, protein-energy wasting, and muscle atrophy, among which chronic systemic inflammation is considered the core driver (32). Dupilumab may improve frailty through several interrelated mechanisms. First, the drug not only suppresses type 2 inflammation but also downregulates proinflammatory cytokines closely linked to frailty, such as IL-6 and TNF-α (33). Second, the marked increases in Treg proportion and IL-10 level observed in our study help restore immune regulation and counteract the proinflammatory bias. Third, by significantly relieving pruritus and improving sleep, dupilumab indirectly promotes physical activity and overall functional status. We are the first to use the Clinical Frailty Scale (CFS) in this population. Our results showed that the CFS score decreased by 17.5% at week 12 (*p* < 0.001) and remained significantly lower than baseline at week 24 (*p* = 0.001), indicating that frailty improves early and the benefit is sustained. This finding expands the recognised benefits of dupilumab beyond skin lesions and pruritus into the domain of global functional status, an outcome of critical importance for patients with advanced CKD. It is important to note that improvements in CFS over 24 weeks may be influenced by symptom relief, particularly the reduction in pruritus and improved sleep, rather than solely representing a reversal of underlying frailty. Whether the observed CFS changes reflect true functional recovery or are primarily mediated by better disease control and symptom alleviation remains to be determined in longer studies with objective functional measures.

Regarding safety, only two patients in this study experienced mild to moderate adverse events, including an injection site reaction and conjunctivitis. Both events resolved with symptomatic management. No patient discontinued treatment because of adverse events, no deaths occurred, and no worsening of renal function was reported. The conjunctivitis rate, approximately 6.9%, was lower than the 10%–20% reported in phase 3 trials (34, 35). In a retrospective analysis of 64 complex patients, including those with chronic kidney disease, Lukac et al. reported that 87.5% improved with dupilumab, the adverse event rate was 20.3%, and the discontinuation rate was 9.4%, indicating a favourable risk-benefit profile even in patients with multiple comorbidities (36). Foti et al. described three AD patients with underlying kidney disease (Alport syndrome, IgA nephropathy, and hypertensive nephrosclerosis), all of whom achieved significant skin improvement without deterioration of renal function (37). Although isolated case reports have suggested that dupilumab might occasionally trigger or worsen glomerular diseases such as IgA nephropathy or lupus-like nephritis (38), these events are rare and were not observed in our study. Importantly, dupilumab is not renally cleared, lacks nephrotoxicity, and has minimal drug-drug interactions. These attributes give it a unique safety advantage in CKD patients, who often require multiple concomitant medications, including antihypertensives and antidiabetic agents.

This study has several limitations. First, the single-centre, retrospective design may introduce selection bias, and the modest sample size precluded subgroup analyses or reliable detection of rare adverse events. Second, the distribution of CKD stages was skewed towards stage 3 (68.97%), with only three stage 5 patients; therefore, extrapolation of these findings to patients with severe renal impairment requires caution. Third, the follow-up period was limited to 24 weeks, leaving longer-term efficacy and safety to be determined. Fourth, the complete-case approach may incur attrition bias because dropouts may systematically differ from completers in response, safety, or severity. Of the 42 patients who initiated dupilumab, 13 (31.0%) did not complete the 24-week follow-up and were excluded from the final analysis; as detailed in Supplementary Table S1, the most common reasons for exclusion were inadequate response (4/13, 30.8%) and adverse events (3/13, 23.1%), followed by loss to follow-up (3/13, 23.1%), patient withdrawal (2/13, 15.4%), and a medical complication related to CKD progression (1/13, 7.7%). Their baseline characteristics are provided in Supplementary Table S1; nevertheless, findings should be interpreted cautiously. Future studies should use intention-to-treat and record dropout reasons. Fifth, the three stage 5 patients were all managed without dialysis; therefore, our safety conclusions do not extend to patients on hemodialysis or peritoneal dialysis, in whom pharmacokinetics and immunological responses may differ.

## 5 Conclusion

In summary, dupilumab demonstrates clear short-term efficacy and acceptable safety in patients with stage 3–5 CKD and moderate-to-severe AD. Beyond improving skin lesions, pruritus, sleep, and quality of life, it shows changes consistent with regulatory immune modulation characterised by increased Treg and IL-10 levels, and also offers early improvement in frailty status. Renal impairment alone should not be considered an absolute contraindication, although patients with advanced CKD require careful monitoring to dupilumab therapy. Future large-scale, prospective, multicentre studies with stratification by CKD stage are warranted to validate our findings and guide individualised treatment.

## Data Availability

The original contributions presented in the study are included in the article/Supplementary Material, further inquiries can be directed to the corresponding author/s.
