# Supplementary material for: Efficacy and safety of dupilumab in moderate-to-severe atopic dermatitis with stage 3–5 chronic kidney disease: a retrospective real-world study
Source: Front Med (Lausanne). 2026 Sep 14;13:1906235. doi: 10.3389/fmed.2026.1906235 (PMC13616674; doi:10.3389/fmed.2026.1906235)
Supplement: Supplementary file 1 [file Table1.docx]

Supplementary Material

# 1 Supplementary Figures and Tables

## 1.1 Supplementary Figures

**Supplementary Figure 1.** Flowchart of inclusion of participants.

**Supplementary Figure 2.** Changes in clinical scores and laboratory parameters in AD patients treated with dupilumab. (A) EASI, (B) Pruritus NRS, (C) Sleep NRS, (D) DLQI, (E) CFS, (F) EOS, (G) Treg, (H) IL-10.

## 1.2 Supplementary Table

#### **Table 1.** Clinical characteristics

| Clinical characteristics | Value (n = 29) |
| --- | --- |
| Sex, male, n (%) | 20 (68.97%) |
| Age, years, Mean ± SD | 55.73 ± 4.28 |
| Age at onset of AD, years, Mean ± SD | 42.31 ± 4.18 |
| Chronic Kidney Disease staging, n (%) |  |
| - Stage 3 (eGFR 30 -59 mL/min/1.73m²) | 20(68.97) |
| - Stage 4 (eGFR 15-29 mL/min/1.73m²) | 6(20.69) |
| - Stage 5 (eGFR <15 mL/min/1.73m²) | 3(10.34) |
| CKD duration, years, M (Q₁, Q₃) | 4.53(2.05, 8.11) |
| CKD etiology |  |
| - Chronic glomerulonephritis | 12(41.38%) |
| - Diabetic nephropathy | 8(27.59%) |
| - Hypertensive nephrosclerosis | 5(17.24%) |
| - Polycystic kidney disease | 4(13.79%) |
| Clinical phenotype, n (%) |  |
| - Generalized type | 10 (34.48%) |
| - Head‑and‑neck type | 7 (24.14%) |
| - Hand eczema type | 5 (17.24%) |
| - Prurigo type | 4 (13.79%) |
| - Erythrodermic type | 3 (10.35%) |
| Nephrology medications |  |
| - Renin-angiotensin system inhibitors | 18 (62.07%) |
| - Sodium-glucose cotransporter-2 inhibitors | 11 (37.93%) |
| - Diuretics | 5 (17.24%) |
| Comorbidities, n (%) |  |
| - Hypertension | 20 (68.97%) |
| - Cardiovascular disease | 17 (58.62%) |
| - Diabetes mellitus | 12 (41.34%) |
| - Chronic obstructive pulmonary disease | 7 (24.14%) |
| - Neurological disorders | 8 (27.59%) |
| Previous systemic therapies, n (%) |  |
| - Antihistamines | 21 (72.41%) |
| - Glucocorticoids | 15 (51.72%) |
| - Traditional Chinese medicine | 10 (34.48%) |
| - Immunosuppressants | 9 (31.03%) |
| - Phototherapy | 6 (20.69%) |
| EASI, Mean±SD | 34.90 ± 7.88 |
| Pruritus NRS, M (Q₁, Q₃) | 8.00 (7.00,9.00) |
| Sleep NRS, M (Q₁, Q₃) | 7.00 (6.00,8.00) |
| DLQI, Mean±SD | 16.38 ± 4.98 |
| CFS, M (Q₁, Q₃) | 4.00 (4.00,5.00) |
| Eosinophil, ×10^9/L, Mean±SD | 0.64 ± 0.20 |
| IL-10, pg/mL, Mean±SD | 14.98 ± 1.88 |
| Treg proportion, %, Mean±SD | 4.35 ± 0.59 |

Eczema Area and Severity Index, EASI; Numeric Rating Scale, NRS; Dermatology Life Quality Index, DLQI; Clinical Frailty Scale, CFS; Regulatory T cell, Treg; Interleukin‑10, IL‑10

**Table 2.** Changes in clinical scores and laboratory parameters in AD patients treated with dupilumab

| Time point | EASI | | Pruritus NRS | | Sleep NRS | | DLQI | | CFS | | EOS, ×10^9/L | | IL-10, pg/mL | | Treg, % | |
| --- | --- | --- | --- | --- | --- | --- | --- | --- | --- | --- | --- | --- | --- | --- | --- | --- |
|  | Mean±SD | P | M (Q₁, Q₃) | P | M (Q₁, Q₃) | P | Mean±SD | P | M (Q₁, Q₃) | P | Mean±SD | P | Mean±SD | P | Mean±SD | P |
| Baseline | 34.90 ± 7.88 |  | 8.00 (7.00,9.00) |  | 7.00 (6.00,8.00) |  | 16.38 ± 4.98 |  | 4.00 (4.00,5.00) |  | 0.64 ± 0.20 |  | 14.98 ± 1.88 |  | 4.35 ± 0.59 |  |
| Week 12 | 19.31 ± 6.09 | 0.043* | 3.00 (3.00,5.00) | 0.000* | 3.00 (2.00,4.00) | 0.000* | 7.86 ± 3.88 | 0.000* | 4.00 (3.00,4.00) | 0.000* | 0.48 ± 0.10 | 0.000* | 22.45 ± 2.42 | 0.000* | 6.56 ± 0.63 | 0.000* |
| Week 24 | 9.38 ± 4.87 | 0.000# | 1.00 (0.00,2.00) | 0.000# | 1.00 (0.00,2.00) | 0.000# | 2.83 ± 2.17 | 0.000# | 2.00 (1.00,3.00) | 0.001# | 0.40 ± 0.08 | 0.000# | 28.27 ± 2.48 | 0.000# | 7.93 ± 0.71 | 0.000# |

*p compared with baseline; #p compared with baseline.

Eczema Area and Severity Index, EASI; Numeric Rating Scale, NRS; Dermatology Life Quality Index, DLQI; Clinical Frailty Scale, CFS; Regulatory T cell, Treg; Interleukin‑10, IL‑10;Eosinophil, EOS.

**Table 3.** Changes in renal function in AD patients treated with dupilumab

| Time point | eGFR (mL/min/1.73m²) | | Serum creatinine (μmol/L) | | UPCR (g/g) | |
| --- | --- | --- | --- | --- | --- | --- |
|  | Mean±SD | P | Mean±SD | P | M (Q₁, Q₃) | P |
| Baseline | 37.93 ± 14.88 |  | 154.90 (124.50, 223.80) |  | 0.802 ± 0.345 |  |
| Week 12 | 37.90 ± 14.97 | 0.920* | 154.00 (125.50, 224.10) | 0.991* | 0.774 ± 0.328 | 0.063* |
| Week 24 | 36.39 ± 14.77 | 0.073# | 160.40 (131.20, 255.30) | 0.130# | 0.765 ± 0.340 | 0.069# |

*p compared with baseline; #p compared with baseline.

Estimated Glomerular Filtration Rate, eGFR; Urine Protein-to-creatinine Ratio, UPCR.

**Supplementary Table 1. Baseline clinical characteristics of the 13 patients who did not complete the 24-week follow-up**

| Characteristic | Value (n = 13) |
| --- | --- |
| Sex, male, n (%) | 8 (61.5%) |
| Age, years, mean ± SD | 59.1 ± 4.3 |
| Age at onset of AD, years, mean ± SD | 40.4 ± 3.5 |
| Chronic Kidney Disease staging, n (%) |  |
| Stage 3 (eGFR 30–59 mL/min/1.73m²) | 6 (46.2%) |
| Stage 4 (eGFR 15–29 mL/min/1.73m²) | 4 (30.8%) |
| Stage 5 (eGFR <15 mL/min/1.73m²) | 3 (23.1%) |
| Estimated GFR, mL/min/1.73m², median (Q₁, Q₃) | 25 (18, 46) |
| Clinical phenotype, n (%) |  |
| Generalized type | 5 (38.5%) |
| Head-and-neck type | 3 (23.1%) |
| Hand eczema type | 2 (15.4%) |
| Prurigo type | 2 (15.4%) |
| Erythrodermic type | 1 (7.7%) |
| Comorbidities, n (%) |  |
| Hypertension | 10 (76.9%) |
| Cardiovascular disease | 8 (61.5%) |
| Diabetes mellitus | 6 (46.2%) |
| Chronic obstructive pulmonary disease | 3 (23.1%) |
| Neurological disorders | 4 (30.8%) |
| Previous systemic therapies, n (%) |  |
| Antihistamines | 10 (76.9%) |
| Glucocorticoids | 7 (53.8%) |
| Traditional Chinese medicine | 5 (38.5%) |
| Immunosuppressants | 4 (30.8%) |
| Phototherapy | 3 (23.1%) |
| EASI, mean ± SD | 36.2 ± 3.9 |
| Pruritus NRS, M (Q₁, Q₃) | 8 (7, 8) |
| Sleep NRS, M (Q₁, Q₃) | 7 (7, 7) |
| DLQI, mean ± SD | 16.1 ± 1.7 |
| CFS, M (Q₁, Q₃) | 4 (4, 5) |
| Eosinophil, ×10⁹/L, mean ± SD | 0.63 ± 0.07 |
| IL-10, pg/mL, mean ± SD | 14.4 ± 0.7 |
| Treg proportion, %, mean ± SD | 4.1 ± 0.3 |
| Follow-up duration, n (%) |  |
| ≤4 weeks | 2 (15.4%) |
| 5–8 weeks | 4 (30.8%) |
| 9–12 weeks | 2 (15.4%) |
| 13–16 weeks | 2 (15.4%) |
| 17–23 weeks | 3 (23.1%) |
| Reason for exclusion, n (%) |  |
| Inadequate response | 4 (30.8%) |
| Adverse event | 3 (23.1%) |
| Lost to follow-up | 3 (23.1%) |
| Patient withdrawal | 2 (15.4%) |
| Medical complication | 1 (7.7%) |

EASI, Eczema Area and Severity Index; NRS, Numeric Rating Scale; DLQI, Dermatology Life Quality Index; CFS, Clinical Frailty Scale; Treg, regulatory T cell; IL-10, interleukin-10; eGFR, estimated glomerular filtration rate; AD, atopic dermatitis; SD, standard deviation; Q₁, first quartile; Q₃, third quartile.

Normally distributed continuous data are presented as mean ± SD; non-normally distributed continuous data are presented as median (Q₁, Q₃). Normality was assessed using the Shapiro–Wilk test.

Stage 3: eGFR 30–59 mL/min/1.73m²; Stage 4: eGFR 15–29 mL/min/1.73m²; Stage 5: eGFR <15 mL/min/1.73m².

Inadequate response was defined as <50% reduction in EASI score from baseline at the last available follow-up visit.

Medical complication refers to CKD progression requiring treatment modification.

**Supplementary Table 2. Absolute Changes (Δ) in clinical scores and laboratory parameters in AD patients treated with dupilumab**

| Variable | Baseline vs Week 12 | | | Baseline vs Week 24 | | |
| --- | --- | --- | --- | --- | --- | --- |
|  | Δ | 95% CI | P | Δ | 95% CI | P |
| EASI | -15.60 | -19.17 to -12.02 | <0.001 | -25.52 | -28.83 to -22.21 | <0.001 |
| Pruritus NRS | -4.00 | -5.01 to -2.99 | <0.001 | -6.72 | -7.40 to -6.05 | <0.001 |
| Sleep NRS | -3.72 | -4.60 to -2.84 | <0.001 | -5.69 | -6.44 to -4.94 | <0.001 |
| DLQI | -8.54 | -10.73 to -6.34 | <0.001 | -13.55 | -15.25 to -11.85 | <0.001 |
| CFS | -0.79 | -1.29 to -0.29 | <0.01 | -2.34 | -2.89 to -1.80 | <0.001 |
| Eosinophil (×10⁹/L) | -0.17 | -0.26 to -0.07 | <0.001 | -0.24 | -0.32 to -0.17 | <0.001 |
| IL-10 (pg/mL) | 7.47 | 6.26 to 8.68 | <0.001 | 13.30 | 12.13 to 14.47 | <0.001 |
| Treg proportion (%) | 2.21 | 1.95 to 2.47 | <0.001 | 3.59 | 3.26 to 3.92 | <0.001 |
| eGFR (mL/min/1.73m²) | -0.03 | -0.67 to 0.60 | 0.920 | -1.55 | -2.63 to -0.47 | <0.01 |
| Serum creatinine (μmol/L) | -0.31 | -6.43 to 5.82 | 0.991 | 7.83 | 0.24 to 15.42 | 0.030 |
| UPCR (g/g) | -0.01 | -0.04 to 0.03 | 0.689 | -0.02 | -0.05 to 0.01 | 0.146 |

Δ = absolute change (follow-up value minus baseline value); CI = confidence interval; EASI = Eczema Area and Severity Index; NRS = Numeric Rating Scale; DLQI = Dermatology Life Quality Index; CFS = Clinical Frailty Scale; eGFR = estimated glomerular filtration rate; UPCR = urine protein-to-creatinine ratio; Treg = regulatory T cell; IL-10 = interleukin-10.

Normally distributed continuous data were analyzed using paired-sample t-tests; non-normally distributed continuous data were analyzed using Wilcoxon signed-rank tests. Serum creatinine was non-normally distributed; all other variables were normally distributed.
